# Receptor of ghrelin is expressed in cutaneous neurofibromas of individuals with neurofibromatosis 1

**DOI:** 10.1186/s13023-017-0734-x

**Published:** 2017-12-20

**Authors:** Rafaela E. Rozza-de-Menezes, Nicolle C. Gaglionone, Raquel M. Andrade-Losso, Orlando H. K. Siqueira, Lilian M. Almeida, Kamila da S. Peruzini, Marco A. C. Guimarães-Filho, Carolina I. Brum, Mauro Geller, Karin S. Cunha

**Affiliations:** 1 Graduate Program in Pathology, School of Medicine, Hospital Universitário Antônio Pedro, Universidade Federal Fluminense, Av. Marquês do Paraná, 303, 4o andar, sala 01 – Centro, Niterói, RJ 24033-900 Brazil; 2 Neurofibromatosis National Center (Centro Nacional de Neurofibromatose), Rio de Janeiro, RJ Brazil; 3 School of Dentistry, Health Institute of Nova Friburgo, Universidade Federal Fluminense, Nova Friburgo, RJ Brazil; 4 Department of General and Specialized Surgery, School of Medicine, Universidade Federal Fluminense, Niterói, RJ Brazil; 5 School of Medicine, Universidade Federal Fluminense, Niterói, RJ Brazil; 6 Department of Pathology, School of Medicine, Universidade Federal Fluminense, Niterói, RJ Brazil; 7 Department of Immunology and Microbiology, School of Medicine, Centro Universitário Serra dos Órgãos (UNIFESO), Teresópolis, RJ Brazil; 8 Instituto de Puericultura e Pediatria Martagão Gesteira, School of Medicine, Universidade Federal do Rio de Janeiro, Rio de Janeiro, RJ Brazil

**Keywords:** Neurofibromatosis 1, Neurofibroma, Ghrelin, Ghrelin receptor

## Abstract

**Background:**

Multiple cutaneous neurofibromas are a hallmark of neurofibromatosis 1 (NF1). They begin to appear during puberty and increase in number and volume during pregnancy, suggesting a hormonal influence. Ghrelin is a hormone that acts via growth hormone secretagogue receptor (GHS-R), which is overexpressed in many neoplasms and is involved in tumorigenesis. We aimed to investigate GHS-R expression in NF1 cutaneous neurofibromas and its relationship with tumors volume, and patient’s age and gender.

**Results:**

Sample comprised 108 cutaneous neurofibromas (55 large and 53 small tumors) from 55 NF1 individuals. GHS-R expression was investigated by immunohistochemistry in tissue micro and macroarrays and quantified using a digital computer-assisted method. All neurofibromas expressed GHS-R, with a percentage of positive cells ranging from 4.9% to 76.1%. Large neurofibromas expressed more GHS-R than the small ones. The percentage of GHS-R-positive cells and intensity of GHS-R expression were positively correlated with neurofibromas volume. GHS-R expression was more common in female gender.

**Conclusions:**

GHS-R is expressed in cutaneous neurofibromas. Larger neurofibromas have a higher percentage of positive cells and higher GHS-R intensity. Based on our results we speculate that ghrelin may have an action on the tumorigenesis of cutaneous neurofibromas. Future studies are required to understand the role of ghrelin in the pathogenesis of NF1-associated cutaneous neurofibroma.

**Electronic supplementary material:**

The online version of this article (10.1186/s13023-017-0734-x) contains supplementary material, which is available to authorized users.

## Background

Multiple cutaneous neurofibromas (cNfs) are a hallmark of neurofibromatosis 1 (NF1). They begin to appear during puberty and increase in number and volume during pregnancy [1–3], suggesting a hormonal influence. Most neurofibromas express progesterone and androgen receptors [4, 5] and in vitro and in vivo studies have shown that neurofibromas grow in the presence of sex hormones [6–8].

Beyond sex hormones, it is possible that other hormones, such as components of growth hormone (GH) axis, could have a role in the pathogenesis of NF1-associated neurofibromas. Most NF1 neurofibromas express GH receptors, which suggests that GH exerts a direct effect on these neoplasms [3, 9]. Ghrelin is another component of GH axis and presents many physiological functions in diverse organs [10]. Ghrelin acts through GH secretagogue receptor (GHS-R) [10]. The classical GHS-R is GHS-R1a, which binds to ghrelin [10]. GHS-R1b is a truncated variant without high-affinity ghrelin binding, with an unclear physiological role. GHS-R1b is expressed in many organs/tissues [11–15], and is also overexpressed in many neoplasms and involved in tumorigenesis [16–22]. Ghrelin promotes cell proliferation of hepatoma [16], pancreatic [21], breast [17], prostate [22] and colon cancers [19].

We aimed to investigate GHS-R expression in NF1 cNfs and its relationship with tumors volume, and patient’s age and gender.

## Methods

Sixty-two individuals with diagnosis of NF1 based on clinical criteria of National Institutes of Health [23] were included in this study. In order to investigate the heterogeneity of GHS-R expression not only in tumors from different individuals but also in tumors from the same individual, each participant had two lesions compatible with cNfs surgically removed: one of the smallest (with at least 4 mm of diameter) and one of his/her largest tumors. Clinically, CNfs were classified as cutaneous if they were limited to the skin and, when moved, the skin over it moves together with the tumor [24]. The neurofibromas volume (expressed in mm^3^) was achieved using ellipsoid volume calculation method (1/2 x Length x Weight x Height) [25].

Samples were handled and processed according to routine histological procedures and a 5-μm haematoxylin/eosin section was used for diagnosis confirmation. We included only non-encapsulated neurofibromas, which represent cNfs [24, 26], and those with immunohistochemical heterogeneous S100 expression (1:100; M7240; Dako Corporation, CA/USA). Subcutaneous neurofibromas, which are confined in an intact perineurium/epineurium (localized intraneural neurofibroma) [26, 27], were excluded since they are different from cNfs not only in their clinical and histopathological aspects but also in terms of prognosis [28, 29].

Tissue microarray (TMA) blocks containing samples from large neurofibromas were constructed as previously described [30], using 1.1 diameter cores from three to five representative regions of each tumor. For small neurofibromas, whole specimens were evaluated after inclusion in tissue macroarrays (TMaAs), using a technique developed by Prof. Dias from UFF (data not published). Briefly, after acquisition of each sample and fixation, histological processing was initiated, stopped after paraffin impregnation, and the specimen was kept in properly identified histological cassette until preparation of the TMaA block. After the collection of all the samples to be included in the TMaA, a thin liquid paraffin layer was placed on a metallic mold and the specimens were attached in an orderly fashion according to a location map. Liquid paraffin was then inserted to construct the paraffin block.

GHS-R was demonstrated immunohistochemically. Negative and positive controls were performed by primary antibody omission and use of normal stomach tissue, respectively. Aperio Digital Pathology® System (Leica Biosystems, Richmond, IL/USA) was used for automatic immunoquantification. Detailed information about immunohistochemistry and immunoquantification procedures is shown in Additional file 1.

Statistical analyses were performed with SPSS v.20 (IBM®). Normality was evaluated with Shapiro-Wilk test. ANOVA one-way (with Bonferroni correction), Student’s *t*-test, paired *t*-test and Pearson’s correlation coefficient were used for variables with normal distribution, and Wilcoxon rank-sum test and Spearman’s correlation coefficient for non-normal distribution variables. Simple linear regression was used to summarize the relationship between the percentage of GHS-R positive cells and volume of tumors. *P*-values ≤0.05 were considered significant.

## Results

Table 1 shows a summary of GHS-R expression data and statistical findings. Additional file 2 (Table S1) and Additional file 3 (Table S2) show clinical data and detailed GHS-R expression data, respectively. After excluding cases without microscopic confirmation of cNf (*n* = 4) and losses during immunohistochemistry (*n* = 12), the sample comprised 55 large and 53 small neurofibromas from 55 participants. All neurofibromas expressed S100. For paired tests, large (*n* = 48) and small tumors (*n* = 48) from the same individuals were considered. Paired values of volume of large versus small neurofibromas were different (*p* < 0.0001, Wilcoxon rank-sum test).

**Table 1:** Summary GHS-R expression data and statistical findings

	Cutaneous Neurofibromas		
	Large	Small	*P-value*
	Mean (± standard deviation)	Mean (± standard deviation)	
Volume (mm^3^)	1923 (±2349)	63.9 (±62.1)	<0.0001^a^
Total number of nuclei per neurofibroma	25,878 (±10,979)	5,2016 (±27,953)	–
Number of positive nuclei per neurofibroma	14,025 (±6310)	21,912 (±14,146)	–
Percent of positive nuclei per neurofibroma/n*	53.6% (±9.2)/48	41.1%(±13.1)/48	<0.0001^b^
Percent of positive nuclei (considering the tumors of the same individuals with volume difference ≥1000 mm^3^)/n*	52.9%(±8.6)/24	44.2%(±14.4)/24	0.01^b^
Percent of positive nuclei (considering the tumors of the same individuals with volume difference of ≥3000 mm^3^)/n*	53.6% (±8.0)/9	35.4% (±16.1)/9	0.015^a^
Percent of cells with strong staining / Number of tumors with predominance of cells with strong staining	24.1%^d^ (±7.1)/42^d^	13.5% (±9)/20	<0.0001^c^
Percent of cells with moderate staining / Number of tumors with predominance of cells with moderate staining	17.9%^d^ (±4.6)/13^d^	13.5% (±4.3)/8	
Percent of cells with weak staining / Number of tumors with predominance of cells with weak staining	11.4% (±2.7)/0 (none)	14.1% (±4.2)/25	
Area of analysis (mm^2^)	4.2 (±1.7)	10.2 (±6.3)	–

^*^ *n*, number of tumors

^a^Wilcoxon rank-sum test

^b^Paired *t*-test

^c^ANOVA one-way (with Bonferroni correction)

^d^values with statistical difference after Bonferroni correction

**Fig. 1:**
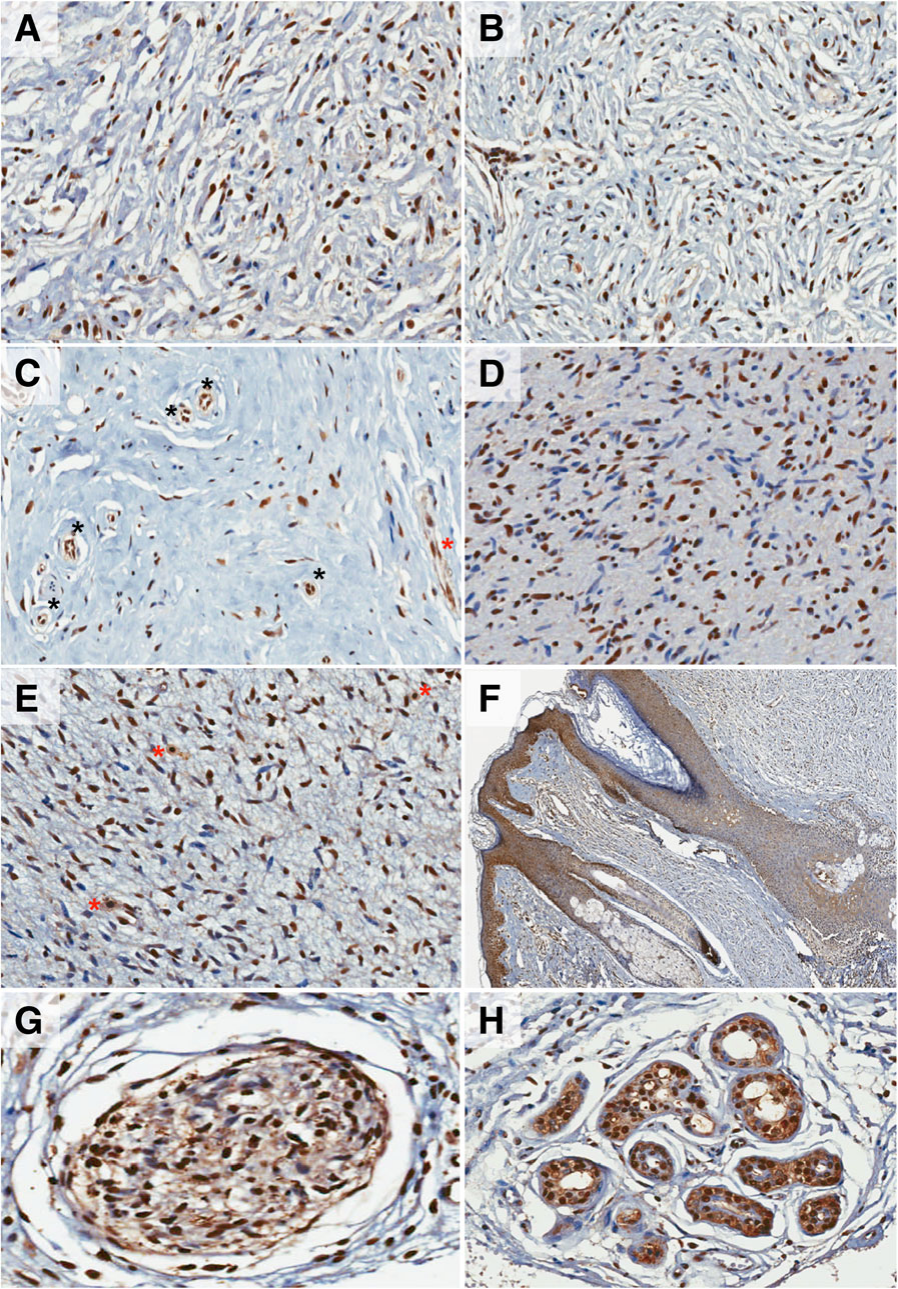
Expression of GHS-R in neurofibromas and other structures. Legend: (**a** − **e**) Neurofibromas expressing GHS-R. Most spindle cells showed nuclear expression, but some also presented cytoplasmic GHS-R expression. (200×); (**c**) Asterisks show GHS-R expression in tumor endothelial cells (nucleus and cytoplasm). Black asterisks: transversal cut of blood vessels. Blue asterisk: longitudinal cut of a blood vessel; (**e**) Asterisks show expression of GHS-R in mast cells. **f** GHS-R expression in epidermis, hair follicles, and sebaceous glands (100×). **g** GHS-R expression in nerve fascicle (400×); (**h**) GHS-R expression in sweat glands (200×)

All neurofibromas expressed GHS-R, which was investigated with an antibody against both GHS-R isoforms. In the tumor area, positivity was seen in fusiform cells (although not all were positive), mast cells, which were identified by their morphology, and endothelial cells of blood vessels (Fig. 1a-e). In the area surrounding the neurofibromas, dermis was negative, but epidermis, skin annexes, nerves, and endothelial cells of blood vessels were positive to GHS-R (Fig. 1f-h).

In the neurofibroma area, the percentage of positive cells to GHS-R varied from 4.9 to 76.1% (mean = 47.5%; ±12.8). Large neurofibromas had a higher percentage of GHS-R-positive cells than small ones (*p* < 0.0001, paired *t*-test). Considering the small and large neurofibromas from the same individuals with volume difference ≥ 1000 mm^3^, the largest had a higher percentage of positive cells (*p* = 0.01, paired *t*-test). Significant statistical differences were also observed when considering neurofibromas with volume difference ≥ 3000 mm^3^ (*p* = 0.015, respectively, Wilcoxon rank-sum test). Considering all tumors, the percentage of GHS-R-positive cells was positively correlated with neurofibromas volume (*p* < 0.0001, Spearman’s correlation coefficient). Linear regression revealed positive correlation between percentage of GHS-R-positive cells and tumors volume (*p* = 0.01). The percentage of positive cells was not related with patient’s age (*p* = 0.62, Pearson’s correlation coefficient), but was positively associated with female gender (*p* = 0.0005, Student’s t-test).

**Fig. 2:**
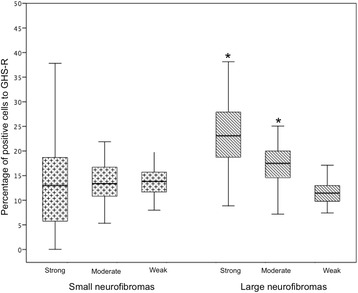
Percentage of GHS-R positive cells according to the staining intensity in small and large neurofibromas of individuals with neurofibromatosis 1. Legend: Large neurofibromas had a higher percentage of moderate/strong staining cells than small neurofibromas (* indicates *p* < 0.0001, ANOVA one-way with Bonferroni correction)

The percentage of cells with weak, moderate and strong GHS-R staining in large and small neurofibromas was significant different (*p* < 0.0001, ANOVA one-way with Bonferroni correction, Fig. 2). Large neurofibromas had a higher percentage of cells with moderate/strong staining than small neurofibromas (*p* < 0.0001, ANOVA one-way with Bonferroni correction, Fig. 2).

## Discussion

We show for the first time that cNfs express GHS-R. Expression of GHS-R was seen in fusiform cells of the tumors. Neurofibromas are composed of Schwann cells [31], as well as perineurial-like cells, fibroblasts, mast cells, and axons [26]. Under optical microscopy, based on their morphology, Schwann cells, perineural-like cells and fibroblasts cannot be distinguished, since all present a fusiform aspect. One limitation of this study was the use of a one staining immunohistochemistry. Other studies are necessary to know which fusiform cells in cutaneous neurofibromas express GHS-R. Mast cells in cNfs also expressed GHS-R and it is known that mast cells are important to neurofibroma tumorigenesis [32]. Future studies are necessary to determine the action of ghrelin on different cells of neurofibromas.

Ghrelin is proangiogenic and normal human endothelial cells express GHS-R [33]. Endothelial cells of blood vessels in the cNfs and also in the surrounding normal dermis were positive to GHS-R. Neurofibromas are highly vascularized and the ghrelin action in promoting their growth by enhancing angiogenesis should be further investigated.

In conclusion, we show that GHS-R is expressed in cNfs. Larger neurofibromas have a higher percentage of positive cells and higher GHS-R intensity. Moreover, GHS-R expression was more common in female gender. Based on our results we speculate that ghrelin may have an action on the tumorigenesis of cNfs. Future studies are required to understand the role of ghrelin in the pathogenesis of NF1-associated cutaneous neurofibroma.

## Additional files

Additional file 1: Supplementary information – Methods. Detailed information about immunohistochemistry and immunoquantification. (PDF 40 kb)

Additional file 2: Table S1. Clinical data of the individuals with neurofibromatosis 1 and neurofibromas included in the study. (PDF 59 kb)

Additional file 3: Table S2. Results of GHS-R expression in neurofibromas. (PDF 79 kb)

## Abbreviations

cNf: Cutaneous neurofibroma
GH: Growth hormone
GHS-R: GH secretagogue receptor
GHS-R1a: GHS-R type 1a
GHS-R1b: GHS-R type 1b
NF1: Neurofibromatosis 1
TMA: Tissue microarray
TMaA: Tissue macroarray
