# Supplementary material for: Receptor of ghrelin is expressed in cutaneous neurofibromas of individuals with neurofibromatosis 1
Source: Orphanet J Rare Dis. 2017 Dec 20;12:186. doi: 10.1186/s13023-017-0734-x (PMC5738781; doi:10.1186/s13023-017-0734-x)
Supplement: Supplementary file 1 — Supplementary information – Methods. Detailed information about immunohistochemistry and immunoquantification. (PDF 40 kb) [file 13023_2017_734_MOESM1_ESM.pdf]

Supplementary information

Methods

Immunohistochemistry

From each paraffin block, 3 µm sections were collected on silane-coated slides. GHS-R was demonstrated immunohistochemically with MACH-4™ (M4BD534H; Biocare Medical, CA/USA). For antigen retrieval, citrate buffer (pH 6.0) in bath water (96°C, 40-min) was used. Endogenous peroxidase activity was eliminated with 3% H_2O_2 in distilled water (room temperature, 30-min). Non-specific protein binding was blocked with normal goat serum (room temperature, 15-min). Sections were incubated (overnight; room temperature) with anti-GHS-R antibody (ab85104; Abcam, MA/USA) in antibody diluent (1:100; S3022; Dako Corporation), stained with diaminobenzidine (3-min) and counterstained with Mayer's haematoxylin.

Immunoquantification

Aperio Digital Pathology® System (Leica Biosystems, Richmond, IL/USA) was used for GHS-R immunoquantification. TMA/TMaA slides were scanned and organized in TMAlab™ software. For analysis, we included only large neurofibromas with ≥2 cores and small neurofibromas with ≥30% of tumor area on the slide.

Tumor area was selected and large blood vessels, inflammatory infiltrate, and artifacts (e.g. tissue folding) were excluded using ScanScope™ software. Immunoquantification was performed with Spectrum™ software through evaluation of the brown (positive) and blue (negative) staining, using Nuclear v.9

algorithm after calibration (in ScanScope™ software). Positive cells percentage (positive cells number/total cells number) and cell percentage with weak, moderate and strong staining were obtained. For the analysis of staining intensity, the ScanScope™ software Nuclear v.9 algorithm was able to classify each pixel as 0 (negative, threshold 256–220), 1 (weak positive staining, threshold 220–175), 2 (moderate positive staning, threshold 175–100), or 3 (strong positive staining, threshold 100–0) and to count the number of pixels in each category. Samples homogeneity was tested through brown/blue ratio.[1] Large ratio variation indicate aberrant spot images, i.e. tissue unspecific diaminobenzidine (brown) deposits.[1]

References

1. Plancoulaine B, Laurinaviciene A, Meskauskas R, Baltrusaityte I, Besusparis J, Herlin P, et al. Digital immunohistochemistry wizard: image analysis-assisted stereology tool to produce reference data set for calibration and quality control. Diagn. Pathol. 2014;9 Suppl 1:S8.
