# Supplementary material for: Receptor of ghrelin is expressed in cutaneous neurofibromas of individuals with neurofibromatosis 1
Source: Orphanet J Rare Dis. 2017 Dec 20;12:186. doi: 10.1186/s13023-017-0734-x (PMC5738781; doi:10.1186/s13023-017-0734-x)
Supplement: Supplementary file 2 — Clinical data of the individuals with neurofibromatosis 1 and neurofibromas included in the study. (PDF 59 kb) [file 13023_2017_734_MOESM2_ESM.pdf]

Table S1: Clinical data of the individuals with neurofibromatosis 1 and neurofibromas included in the study

Case	Age (years)	Sex	**Large neurofibroma** (n=55)		**Small neurofibroma** (n=53)	
			**Site**	**Volume (mm³)**	**Site**	**Volume (mm³)**
1	46	Female	Upper limb	336	Trunk	32
2	45	Male	Lower limb	2,184	Upper limb	SE*
3	66	Female	Head and neck	780	Head and neck	375
4	71	Female	Lower limb	1,275	Head and neck	SE*
5	35	Female	Head and neck	SE*	Head and neck	SE*
6	23	Female	Trunk	2,295	Trunk	SE*
7	52	Female	Lower limb	1,035	Trunk	70
8	46	Female	Lower limb	2,800	Trunk	70
9	35	Male	Trunk	900	Trunk	40
10	43	Male	Upper limb	10,395	Lower limb	96
11	51	Male	Trunk	2,200	Trunk	40
12	44	Female	Trunk	600	Trunk	40
13	54	Female	Trunk	1,485	Head and neck	48
14	67	Female	Upper limb	144	Lower limb	64
15	37	Female	Lower limb	4,200	Lower limb	96
16	58	Female	Trunk	168	Trunk	72
17	31	Female	Upper limb	2,268	Trunk	87.5
18	26	Female	Lower limb	5,250	Trunk	140
19	24	Female	Trunk	210	Trunk	MN‡
20	49	Female	Trunk	5,060	Trunk	140
21	40	Female	Lower limb	520	Trunk	160
22	56	Female	Trunk	1,482	Lower limb	62.5
23	45	Female	Lower limb	8,835	Lower limb	SE*
24	55	Female	Lower limb	1,912	Lower limb	80
25	51	Male	Trunk	2,025	Trunk	126
26	55	Female	Trunk	3,000	Trunk	210
27	66	Male	Lower limb	5,000	Upper limb	168
28	54	Female	Lower limb	3,887	Lower limb	36
29	44	Female	Lower limb	171	Upper limb	108
30	37	Female	Trunk	3,150	Head and neck	75
31	23	Female	Lower limb	28	Upper limb	12.5
32	32	Female	Trunk	55	Trunk	32
33	31	Male	Trunk	380	Trunk	32
34	39	Female	Trunk	162	Head and neck	SE*
35	37	Female	Lower limb	144	Trunk	40
36	16	Male	Trunk	17.5	Trunk	7.5
37	13	Female	Lower limb	1,155	Trunk	8
38	37	Male	Upper limb	1694	Upper limb	30
39	42	Female	Head and neck	245	Head and neck	SE*
40	47	Male	Upper limb	8,208	Head and neck	18
41	45	Female	Trunk	325	Trunk	12
42	68	Male	Upper limb	429	Head and neck	70
43	43	Male	Lower limb	660	Head and neck	84
44	61	Female	Upper limb	858	Upper limb	40
45	25	Female	Upper limb	420	Upper limb	20
46	54	Male	Upper limb	1,242	Trunk	24
47	56	Female	Trunk	35	Trunk	12
48	24	Female	Lower limb	1,568	Trunk	20
49	29	Female	Lower limb	2,835	Head and neck	35
50	32	Female	Trunk	742.5	Head and neck	40
51	39	Female	Trunk	180	Upper limb	62.5
52	62	Female	Trunk	2,025	Head and neck	60
53	77	Female	Lower limb	6,930	Trunk	60
54	23	Male	Lower limb	PN†	Trunk	24
55	41	Female	Trunk	210	Trunk	32
56	28	Female	Lower limb	180	Trunk	36
57	48	Female	Lower limb	PN†	Trunk	36
58	30	Male	Lower limb	SE*	Trunk	24
59	37	Female	Trunk	1495	Trunk	20
60	22	Male	Upper limb	SE*	Head and Neck	H§
61	48	Female	Lower limb	SE*	Trunk	40
62	17	Female	Lower limb	SE*	Trunk	15

*SE, sample excluded (lost during immunohistochemical technique); †PN, plexiform neurofibroma; ‡MN, melanocytic nevus; §H, hemangioma. Note. Tumor volume was achieved using ellipsoid volume calculation method (1/2 x Length x Weight x Height)[26] and was expressed in mm³.
