# Supplementary material for: Receptor of ghrelin is expressed in cutaneous neurofibromas of individuals with neurofibromatosis 1
Source: Orphanet J Rare Dis. 2017 Dec 20;12:186. doi: 10.1186/s13023-017-0734-x (PMC5738781; doi:10.1186/s13023-017-0734-x)
Supplement: Supplementary file 3 — Results of GHS-R expression in neurofibromas. (PDF 79 kb) [file 13023_2017_734_MOESM3_ESM.pdf]

Table S2: Results of immunostaining quantification of ghrelin receptor in neurofibromas

Case	Tumor	Total Nuclei	Positive Nuclei	Percent of positive nuclei	Percent of nuclei with strong immunostaining	Percent of nuclei with moderate immunostaining	Percent of nuclei with weak immunostaining
1	Large	10,906	5,230	47.9%	17.3%	19.2%	11.4%
	Small	11,547	3,591	31.1%	4%	10.6%	16.5%
2	Large	20,836	11,353	54.4%	21.1%	21.1%	12.2%
	Small	SE†	SE†	–	–	–	–
3	Large	26,430	13,305	50.3%	8.8%	26.1%	15.4%
	Small	45,322	15,638	34.5%	9.2%	11.4%	13.8%
4	Large	37,244	18,636	50%	25.7%	13.6%	10.7%
	Small	SE†	SE†	–	–	–	–
5	Large	SE†	SE†	–	–	–	–
	Small	SE†	SE†	–	–	–	–
6	Large	45,414	24,761	54.5%	26.8%	16.0%	11.7%
	Small	SE†	SE†	SE†	–	–	–
7	Large	17,993	8,764	48.7%	26.9%	12.8%	9.0%
	Small	72,198	31,040	42.9%	17%	14.2%	11.7%
8	Large	19,902	9,305	46.7%	25.9%	13.4%	7.4%
	Small	51,541	28,693	55.6%	23.8%	18.7%	13.1%
9	Large	28,833	12,643	43.8%	14.5%	16.1%	13.2%
	Small	67,595	28,412	42%	13.8%	14.1%	14.1%
10	Large	35,662	18,323	51.3%	21%	17.5%	12.8%
	Small	28,653	7,256	25.3%	2.2%	7.6%	15.5%
11	Large	23,493	11,240	47.8%	22.9%	14.2%	10.7%
	Small	48,243	24,314	50.4%	20.2%	17.2%	13.0%
12	Large	27,236	16,112	59.1%	32.2%	15.9%	11.0%
	Small	44,012	19,411	44.1%	21.1%	13.0%	10.0%
13	Large	18,550	7,959	42.9%	23.2%	12.2%	7.5%
	Small	44,899	23,986	53.4%	26%	16.3%	11.1%
14	Large	22,619	13,340	58.9%	27.3%	17.4%	11.6%
	Small	27,172	9,293	34.2%	12.5%	11.0%	10.7%
15	Large	8,298	3,709	44.6%	24%	12.0%	8.6%
	Small	30,574	2,658	8.6%	0	0.4%	8.2%
16	Large	2,085	484	23.2%	12.9%	7.1%	3.2%
	Small	33,181	1,657	4.9%	0	0.5%	4.4%
17	Large	25,425	12,326	48.4%	21.5%	15.5%	11.4%
	Small	69,283	22,292	32.1%	1.8%	10.5%	19.8%
18	Large	38,842	16,539	42.5%	21%	11.8%	9.7%
	Small	56,732	13,059	23%	1.5%	7.1%	14.4%
19	Large	10,081	4,956	49.1%	18.6%	17.5%	13.0%
	Small	MN‡	MN‡	–	–	–	–
20	Large	57,388	33,627	60.1%	16.2%	24.5%	19.3%
	Small	38,587	16,573	42.9%	12%	16.5%	14.4%
21	Large	21,010	9,475	49%	18.8%	14.9%	11.9%
	Small	131,497	27,930	21.2%	3.2%	6.2%	11.8%
22	Large	23,483	18,078	76.1%	46.6%	18.9%	10.6%
	Small	50,979	25,824	50.6%	4.6%	18.8%	27.2%

**23**	Large	36,148	25,267	69.8%	28%	25.0%	16.8%
	Small	SE†	SE†	–	–	–	–
**24**	Large	30,552	17,036	55.7%	24.7%	17.7%	13.3%
	Small	158,164	84,386	53.3%	17.3%	20.0%	16.0%
**25**	Large	38,121	21,546	56.5%	31.5%	15.7%	9.3%
	Small	38,940	14,945	38.3%	11.9%	12.7%	13.7%
**26**	Large	39,872	18,831	47.2%	19.4%	16.5%	11.3%
	Small	26,409	18,098	68.5%	37.8%	18.4%	12.3%
**27**	Large	31,048	16,455	52.9%	25.1%	17.4%	10.4%
	Small	50,570	32,641	64.5%	27.2%	21.8%	15.5%
**28**	Large	29,654	19,919	67.1%	45%	13.5%	8.6%
	Small	9,601	4,671	48.6%	19.6%	15.5%	13.5%
**29**	Large	27,879	17,914	64.2%	22.4%	26.1%	15.7%
	Small	60,640	31,123	51.3%	17.5%	18.0%	15.8%
**30**	Large	19,290	9,075	47%	25.2%	12.4%	9.4%
	Small	47,220	19,256	40.7%	5.7%	15.3%	19.7%
**31**	Large	26,618	15,605	58.6%	24.9%	19.5%	14.2%
	Small	58,670	30,836	52.5%	18.5%	18.3%	15.7%
**32**	Large	31,071	17,435	56.1%	23.1%	20.4%	12.6%
	Small	33,539	15,076	44.9%	7.4%	15.4%	22.1%
**33**	Large	26,110	13,903	53.2%	33.5%	12.1%	7.6%
	Small	26,037	14,419	55.3%	25.3%	17.2%	12.8%
**34**	Large	34,160	19,721	57.7%	18.4%	24.0%	15.3%
	Small	SE†	SE†	–	–	–	–
**35**	Large	34,643	22,227	64.1%	36.7%	17.0%	10.4%
	Small	38,660	14,461	37.4%	12.4%	12.4%	12.6%
**36**	Large	34,810	17,244	49.5%	24.9%	13.3%	11.3%
	Small	19,190	5,190	27%	6.1%	9.4%	11.5%
**37**	Large	11,695	5,840	49.9%	31.3%	10.3%	8.3%
	Small	53,547	26,917	50.2%	15.8%	19.0%	15.4%
**38**	Large	41,088	16,749	40.7%	12.5%	15.2%	13.0%
	Small	40,571	20,172	49.7%	24.3%	15.0%	10.4%
**39**	Large	27,903	14,694	52.6%	15.6%	19.9%	17.1%
	Small	SE†	SE†	–	–	–	–
**40**	Large	42,393	19,810	46.7%	18.3%	15.2%	13.2%
	Small	32,816	10,604	32.3%	5.2%	11.1%	16.0%
**41**	Large	28,482	15,088	52.9%	18%	22.0%	12.9%
	Small	50,064	19,403	38.7%	11%	13.3%	14.4%
**42**	Large	19,938	8,064	40.4%	13.4%	15.2%	11.8%
	Small	62,233	35,589	57.1%	19.6%	20.7%	16.8%
**43**	Large	24,327	12,255	50.3%	20.1%	18.1%	12.1%
	Small	54,741	18,555	33.9%	9.3%	10.8%	13.8%
**44**	Large	35,777	22,401	62.6%	26.4%	23.9%	12.3%
	Small	52,917	23,804	44.9%	13%	16.0%	15.9%
**45**	Large	25,958	14,970	57.6%	23.6%	22.2%	11.8%
	Small	33,387	12,339	36.9%	9%	12.9%	15.0%
**46**	Large	20,224	9,398	46.4%	16.7%	19.2%	10.5%
	Small	58,276	27,057	46.4%	17.7%	15.1%	13.6%

**47**	Large	8,618	4,808	55.7%	29%	17.5%	9.2%
	Small	39,498	13,288	33.6%	13.3%	10.7%	9.6%
**48**	Large	26,072	13,576	52%	22.2%	20.1%	9.7%
	Small	77,110	37,221	48.2%	29.2%	11.0%	8.0%
**49**	Large	15,206	8,997	59.1%	29.8%	19.8%	9.5%
	Small	114,671	55,090	48%	16.7%	16.7%	14.6%
**50**	Large	15,492	9,638	62.2%	26.9%	23.9%	11.4%
	Small	35,511	16,746	47.1%	4%	15.7%	27.4%
**51**	Large	2,702	1,542	57%	26.9%	19.0%	11.1%
	Small	50,028	12,806	25.6%	2.6%	8.0%	15.0%
**52**	Large	20,719	14,133	68.2%	29.4%	24.5%	14.3%
	Small	48,316	32,811	67.9%	33.6%	21%	13.3%
**53**	Large	15,483	9,192	59,3%	31%	18.9%	9.4%
	Small	97,642	32,391	33.1%	7.1%	11.0%	15.0%
**54**	Large	PN§	PN§	–	–	–	–
	Small	85,930	40,464	47%	25.5%	12.5%	9.0%
**55**	Large	20,925	14,125	67.5%	23%	30.0%	14.5%
	Small	55,770	26,881	48.2%	13.3%	17.1%	17.8%
**56**	Large	18,788	13,812	73.5%	38.4%	24.4%	10.7%
	Small	46,292	18,566	40.1%	17.6%	11.8%	10.7%
**57**	Large	PN§	PN§	–	–	–	–
	Small	64,672	30,017	46.4%	9.2%	16.5%	20.7%
**58**	Large	SE†	SE†	–	–	–	–
	Small	27,025	4,841	17.9%	2.2%	5.3%	10.4%
**59**	Large	39,847	20,014	50.7%	23.8%	17.0%	9.9%
	Small	35,123	10,635	30.2%	3.1%	9.1%	18.0%
**60**	Large	SE†	SE†	–	–	–	–
	Small	H¶	H¶	–	–	–	–
**61**	Large	SE†	SE†	–	–	–	–
	Small	96,347	39,007	40.4%	16.8%	12.0%	11.6%
**62**	Large	SE†	SE†	–	–	–	–
	Small	24,744	9,443	38.1%	18.6%	10.5%	9.0%

*Area of analysis in mm^2; SE†, sample excluded (lost during immunohistochemical technique); MN‡, Melanocytic nevus; PN§, Plexiform neurofibroma; H¶, Hemangioma.
